# Trends in global health research among universities in China: a bibliometric analysis

**DOI:** 10.1186/s41256-023-00295-1

**Published:** 2023-04-06

**Authors:** Chenkai Wu, Lijing L. Yan, Qian Long, Yunguo Liu, Jie Tan, Zhexun Lou, Shenglan Tang

**Affiliations:** 1 Global Health Research Center, Duke Kunshan University, Academic Building 3038, No. 8 Duke Avenue, Kunshan, 215316 Jiangsu China; 2 Duke Global Health Institute, Duke University, Durham, NC USA; 3 School of Health Sciences, Wuhan University, Wuhan, Hubei China

**Keywords:** China, Global health, Health governance, Non-communicable disease, Maternal and child health

## Abstract

**Background:**

There has been considerable progress in developing global health education and research in China. Nevertheless, evidence of the progress of Chinese universities’ contributions to global health research is limited. More efforts are needed to depict the progress Chinese universities have collectively made in advancing the field of global health. This study aimed to examine Chinese universities’ collective contributions to global health research by describing the longitudinal trends in global health research publications, uncovering research themes in global health, and exploring collaboration patterns.

**Methods:**

A comprehensive bibliometric analysis was conducted for original research studies of the ten founding members of the China Consortium of Universities for Global Health, one of the largest networks of global health research and education in China.

**Results:**

We found that (1) the number of research publications in the field of global health has steadily increased from 2014 to 2020, (2) non-communicable disease was the most popular research topic, accounting for over one-third of total publications, followed by maternal and child health and neurological and mental disorders and diseases, (3) less than one-fifth of papers involved primary data collection, with the majority of the study populations from low-income and lower-middle-income countries in Asia and Africa, and (4) a sizable collaboration network has been established with co-authors from over 200 oversea universities or organizations, with about one third from the US.

**Conclusions:**

Despite a variety of challenges and barriers, Chinese universities have been playing an increasingly important role in global health research as assessed by peer-reviewed publications over the last decade. More concerted efforts by multiple stakeholders, including government, private sectors, funding agencies, academic institutions, and researchers, are needed to advance the development of global health research in China.

**Supplementary Information:**

The online version contains supplementary material available at 10.1186/s41256-023-00295-1.

## Introduction

In 1963, the Chinese government dispatched its first medical team to Algeria, providing health care services to rural and suburban communities. This initiative, which later evolved into a signature international health aid program, marked the beginning of China’s participation in global health in modern times. Owing to the rapid economic development since the reform in 1978, China has been playing an increasingly active and essential role in global health; the focus was bilateral health aid provided in five major ways: deployment of medical teams, construction of medical facilities, donation of drugs and medical equipment, training of healthcare personnel, and infectious disease control [1]. This process was accelerated by the severe acute respiratory syndrome outbreak in 2003 [2]. Since then, along with its rapidly growing economy, China has substantially increased its financial investment in global health and diversified its collaboration mechanisms. In recent years, the Chinese government has made firm commitments to global health and set an ambitious agenda for promoting health globally through strengthening multilateral collaborations, such as South-South cooperation, the Health Silk Road, and the China-Africa health cooperation. In 2018, the China International Development Cooperation Agency was founded. The establishment of this agency was a milestone in China’s foreign aid history, marking a transition from bilateral aid as the primary form to broader international development cooperation [3].

As China becomes increasingly involved in global health, the biggest challenge is enhancing the quality and broadening the scope of its global health engagement [4]. Building a global health discipline and education programs in Chinese universities and establishing research institutions focusing on global health are essential components of such a grand plan. Since the twenty-first century, we have witnessed considerable progress in developing global health education and research in China. In 2007, Peking University took the lead in launching the first global health institution in China, followed 5 years later by the first global health degree program at Wuhan University and a master's degree program at Duke Kunshan University jointly with Duke University in 2013 [5, 6]. These actions have kept the momentum. In November 2013, the China Consortium of Universities for Global Health (CCUGH) was launched by 10 founding universities—Central South University, Chinese University of Hong Kong, Duke Kunshan University, Fudan University, Kunming University, Peking University, Peking Union Medical College, Sun Yat-sen University, Wuhan University, and Zhejiang University—aiming to promote global health education, training, and research in Chinese universities through sharing of resources, knowledge, and experience, fostering of international and domestic partnerships, the establishment of field sites, and provision of social services [1]. The CCUGH has expanded rapidly, growing from ten founding members to 29 by 2021. Due to several driving factors, such as government’s investment in research, enhanced research capacity of Chinese scholars, and expanding international collaboration, there has been a proliferation of research publications in various global health areas [7–9].

In a recent paper, Kwete et al. [10] reviewed the research capacity of global health institutions in China, focusing on several key quantitative indicators, such as scholarly publications, research grants, faculty members, course curricula, and trainees. Other studies highlighted the opportunities and potential for global health research in China and identified several challenges [6, 11–14]. Nevertheless, evidence of the progress of Chinese universities’ contributions to global health research is generally limited. Evaluation of the trends in research output has been lacking. More efforts are needed to depict the progress Chinese universities have collectively made in advancing the field of global health, which is critical for identifying the strengths, weaknesses, opportunities, and challenges to future global health research in China. To this end, we conducted a bibliometric analysis to assess Chinese universities’ contributions to global health and their research capacity, primarily focusing on describing the emerging trends in global health research publications for studies conducted, at least partially, outside the national borders of China, uncovering major research themes in global health, and exploring collaboration patterns. The findings of this work would help identify the progress and status of global health research among Chinese universities and advance our understanding of opportunities and challenges for Chinese scholars to conducting global health research in the future.

## Methods

### Inclusion and exclusion criteria

Global health is a versatile concept, and a consensus on a precise definition is lacking. Different scholars have proposed many definitions of global health over the past decades [15]. We adopted a widely recognized working definition of global health [16], focusing on transnational health issues, determinants, and solutions. Studies were included based on the following criteria: (i) original research articles published in English; we focused on English-language publications because researchers in Chinese universities mainly submit high-quality manuscripts to English-language journals primarily due to the performance evaluation system’s strong preference on SCI (Science Citation Indexed) papers, (ii) published in a peer-reviewed journal in the period from January 2014 to December 2020 (the CCUGH was established in November 2013), (iii) at least one study population (i.e., location of data collection) is not from China (including China mainland, Taiwan, Hong Kong, and Macao); we adopted a more inclusive approach to include studies that are technically international health research, which focuses on bilateral efforts on health issues in less developed areas, because the term “international health” has been rarely used in China, (iv) the primary affiliation of first or corresponding author (including joint first or corresponding authors) is one of the ten founding members of the CCUGH; these categories are conventionally associated with the highest amount of contribution and reputation [17]. The academic evaluation system in China has a long tradition of valuing primary affiliation and first/corresponding author of scholarly works; authorship other than the first and corresponding author is generally undervalued. We focused on the ten founding members of the CCUGH because these institutions are the pioneers of global health research in China. The member institutions that later joined the CCUGH are still in the nascent stage of developing research capacity in global health; the time to capture their research contributions was shorter and differed from the ten founding members. We excluded meta-analyses, reviews, and studies on research methodology and non-human research.

### Search strategies and data extraction

We conducted an extensive electronic literature search on PubMed and EMBASE from January 2014 to December 2020 for articles written in English. Because global health encompasses a wide variety of thematic areas on health issues, conducting a traditional keyword search in the Title and Abstract fields was difficult to identify eligible articles. Alternatively, we adopted an affiliation-based search strategy to identify articles published by the specific college, school, department, institute, or center of global health/public health within each founding member of the CCUGH. We did not restrict the search to global health institutions because most China-based researchers conducting global health research have a primary affiliation with a public health institution. The search strategy is available in Additional file 1.

Two trained abstractors (JT & ZL) screened the titles, abstracts, and author information for inclusion based on our criteria, followed by a detailed abstract and full-text review. Disagreements and discrepancies were resolved through discussion between two abstractors and a senior researcher of the study team (CW). JT, ZL, and CW subsequently coded eligible articles in an Excel sheet, recording information on year of publication, journal, author names and affiliations, the origin of the study population (countries and continents), article type (primary data collection vs. secondary data analysis), and research topic. We classified eligible articles according to the list of global health research topics (18 categories) established by the Fogarty International Center at the US National Institute of Health [18]. Health policy and systems research was included as an additional topic because it was one of the research priorities in the CCUGH [1]. We classified each eligible article into one research topic; we used the most relevant one for papers focusing on two or more topics. Any disagreement was resolved through group discussion, during which a holistic evaluation was conducted based on the paper’s primary research goal, research expertise of the first/corresponding author, and the journal’s aim and scope.

### Analytic approach

The goal of the present study was to characterize the patterns and trends in global health research among universities in China. Therefore, we presented aggregated data over all founding members of the CCUGH instead of university-specific data. We calculated the total number of articles by year of publication (2014–2020), research topic, and origin of the study population (region [Asia, Africa, Europe, North America, Oceania, and South America] and country). Analyses were stratified by income level (low-income, lower-middle-income, upper-middle-income, and high-income) according to the latest classification of country income groups by the World Bank [19]. Linear trend analysis was used to determine whether the number of publications changed significantly over time. In addition, we counted the number of overseas collaboration institutions (i.e., not in China mainland, Taiwan, Hong Kong, or Macao) by country; only primary affiliations were included. We classified these collaboration institutions as universities and others (including government agencies, hospitals, non-governmental organizations, or international organizations). Moreover, we counted the number of papers with authors from oversea institutions and those with only authors from Chinese institutions. Statistical analyses were conducted using Stata 15.0 and Microsoft Excel.

## Results

### Year and topic of publications

**Fig. 1:**
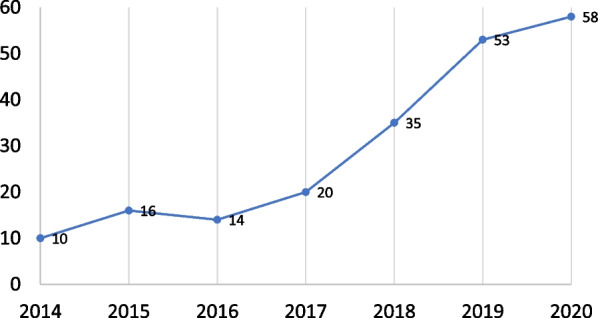
Yearly peer-reviewed publications with data from outside of China by ten founding members of the Chinese Consortium of Universities for Global Health from 2014 to 2020. *Notes* Ten founding members of the Chinese Consortium of Universities for Global Health are (in alphabetical order): Central South University, Duke Kunshan University, Fudan University, Kunming Medical University, Peking University, Peking Union Medical College, Sun Yat-sen University, The Chinese University of Hong Kong, Wuhan University, and Zhejiang University. *P-value for trend* < *.001*

**Fig. 2:**
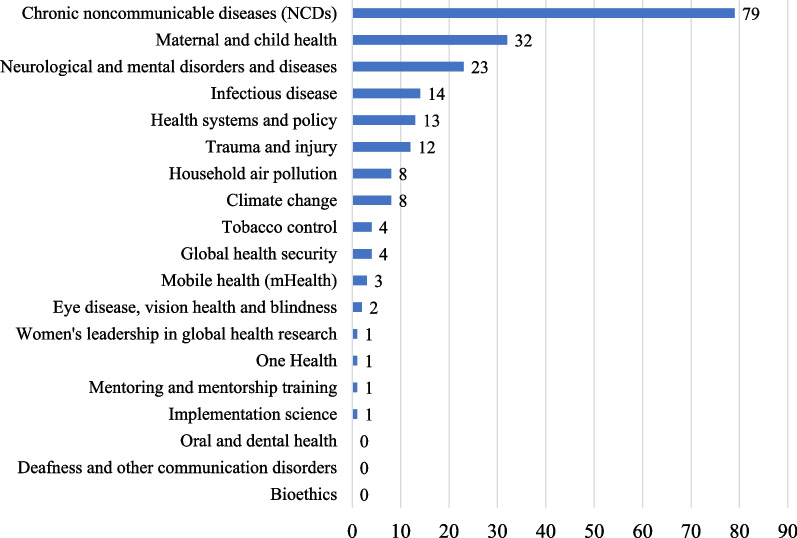
Number of peer-reviewed papers by global health research topic. *Notes* The classification is based on the Fogarty International Center Global Health Research Topics: https://www.fic.nih.gov/researchtopics. Health systems and policy, one of the Chinese Consortium of Universities for Global Health research priorities, is also included as a research topic

The search strategy retrieved 12,542 potentially relevant records from PubMed (n = 10,611) and EMBASE (n = 11,433) after removing duplicates. Based on the title and abstract screening, 206 papers were considered eligible; 10, 16, 14, 20, 35, 53, and 58 papers were published in 2014, 2015, 2016, 2017, 2018, 2019, and 2020, respectively (Fig. 1). Linear trend analysis revealed a significantly increasing trend of publications from 2014 to 2020 (*P-value for trend* < *0.001*). Figure 2 shows the number and relative frequency of these eligible publications by research topic. The number of articles was highest for NCDs (n = 78, 37.9%), followed by maternal and child health (MCH; n = 32, 15.5%), neurological and mental disorders and diseases (n = 23, 11.2%), infectious disease (n = 14, 6.8%), health systems and policy (n = 13, 6.3%), and trauma and injury (n = 12, 5.8%). The number of articles for the other 13 topics was no more than eight (3.9%).

## Research type

Thirty-four (16.5%) papers involved primary data collection, and 172 (83.5%) papers used secondary data analysis. Of 34 papers using primary data collection, 17 (50.0%), 12 (35.3%), 3 (8.8%), 1 (2.9%), and 1 (2.9%) had study populations from Asian, African, North American, European, and Oceanian countries, respectively; 6 (17.6%), 21 (61.8%), 3 (8.8%), and 4 (11.8%) involved study populations in low-income, lower-middle-income, upper-middle-income, and high-income countries, respectively (Table 1). Of 172 papers using secondary data analysis, 44 (25.6%) had study populations from countries (excluding China) of at least two continents; 30 (17.4%), 6 (3.5%), 15 (8.7%), 72 (41.9%), and 5 (2.9%) had study populations in Asia, Africa, North America, Europe, and Oceania, respectively.

**Table 1:** The number of papers by region and country

	Primary data collection	Secondary data analysis	Total
Multi-region	0	44^a^	44
Asia	17	30^b^	47
Lower-middle-income			
Myanmar	5	0	5
Nepal	4	0	4
Vietnam	2	2	4
Bangladesh	2	0	2
India	1	0	1
Pakistan	1	0	1
Timor-Leste	0	1	1
Iran	0	1	1
Upper-middle-income			
Malaysia	2	0	2
Thailand	0	1	1
High-income			
Japan	0	4	4
Singapore	0	2	2
Africa	12	6^c^	18
Low-income			
Malawi	4	0	4
Mozambique	1	1	2
Eritrea	1	0	1
Lower-middle-income			
Tanzania	2	2	4
Zambia	2	0	2
Ghana	1	0	1
Kenya	1	0	1
Europe	1	13^d^	14
High-income			
Denmark	0	2	2
Sweden	0	2	2
Ireland	1	0	1
North America	3	72	77
Upper-middle-income			
Mexico	0	2	2
High-income			
USA	3	69	72
Canada	0	3	3
Oceania	1	5	6
Upper-middle-income			
Republic of Vanuatu	1	0	1
High-income			
Australia	0	5	5
South America	0	0	0

^a^Including research with study populations from countries (not including China mainland, Taiwan, Hong Kong, or Macao) of at least two continents

^b^Including research with study populations from at least two countries (not including China mainland, Taiwan, Hong Kong, or Macao) in Asia

^c^Including research with study populations from at least two countries in Africa

^d^Including research with study populations from at least two countries in Europe

### Collaboration institutions

The global geographical distribution of overseas collaboration institutions is displayed in Table 2, revealing a high concentration of institutions in coastal regions. Overall, there were 223 overseas collaboration institutions, including 159 (71.3%) universities and 64 (28.7%) government agencies, hospitals, non-governmental organizations, or international organizations (Table 2). North America had the highest number of collaboration institutions (n = 80, 35.9%), followed by Asia (n = 65, 29.1%), Europe (n = 36, 16.1%), Africa (n = 17, 7.6%), Oceania (n = 15, 6.7%), and South America (n = 6, 2.7%). Four (1.8%), 37 (16.6%), 21 (9.4%), and 157 (70.4%) collaboration institutions were in low-income, lower-middle-income, upper-middle-income, and high-income countries, respectively. The number of collaboration institutions was highest in the US (n = 64, 28.7%), followed by Japan (n = 21, 9.4%), Australia and Canada (n = 13, 5.8%), and India and UK (n = 11, 4.9%). A total of 163 (79.1%) papers had co-authors from overseas collaboration institutions. Among these papers, 36 (17.5%), 33 (16.0%), 24 (11.7%), 21 (10.2%) and 49 (23.8%) had co-authors from one, two, three, four, and five or more countries, respectively.

**Table 2:** Number of overseas collaboration institutions by country

	Countries	Universities	Others	Total
	*Lower-middle-income*			
Asia Total: 65	Bangladesh	2	1	3
	Cambodia	0	1	1
	India	3	8	11
	Pakistan	3	1	4
	Vietnam	3	1	4
	Iran	2	0	2
	Myanmar	0	1	1
	Philippines	1	0	1
	*Upper-middle-income*			
	Malaysia	5	1	6
	Thailand	0	1	1
	*High-income*			
	Japan	14	7	21
	Singapore	1	3	4
	South Korea	4	0	4
	Saudi Arabia	1	0	1
	United Arab Emirates	0	1	1
Africa Total: 17	*Low-income*			
	Malawi	1	2	3
	Mozambique	1	0	1
	*Lower-middle-income*			
	Tanzania	2	2	4
	Kenya	2	0	2
	Egypt	1	0	1
	Ghana	1	0	1
	Nigeria	1	0	1
	Zambia	1	0	1
	*Upper-middle-income*			
	South Africa	3	0	3
	*Upper-middle-income*			
	Turkey	1	0	1
	*High-income*			
Europe Total: 36	UK	10	1	11
	Sweden	5	0	5
	Denmark	2	2	4
	Netherlands	2	1	3
	Ireland	2	0	2
	Finland	0	2	2
	Belgium	1	0	1
	France	0	1	1
	Germany	0	1	1
	Greece	1	0	1
	Italy	0	1	1
	Norway	1	0	1
	Poland	1	0	1
	Spain	0	1	1
	*Upper-middle income*			
North America Total: 80	Mexico	0	3	3
	*High-income*			
	USA	55	9	64
	Canada	11	2	13
	*Upper-middle-income*			
Oceania Total: 15	Republic of Vanuatu	0	2	2
	*High-income*			
	Australia	11	2	13
	*Upper-middle-income*			
	Argentina	0	2	2
South America Total: 6	Colombia	1	1	2
	Peru	1	0	1
	*High-income*			
	Chile	1	0	1
Others^a^		0	1	1
Total		159	64	223

^a^World Health Organization was a co-author’s first affiliation for one peer-reviewed paper

## Discussion

Between 2014 and 2020, the number of research publications in the field of global health has steadily increased since the establishment of the CCUGH. Only 10 papers were published in 2014, whereas this number increased to 58 in 2020. These results were consistent with previous research showing an increasing trend in publication records for global health institutions in China [10]. We noticed discrepancies in the number of publications between the two studies. This is primarily due to the differences in search strategies and inclusion criteria between the two studies. One major difference was that we only included articles whose first or corresponding authors were affiliated with eligible institutions. Our rationale is that these categories, which are conventionally associated with the highest contribution and reputation [17], more accurately reflect the research capacity. Although the research output in global health has increased, the overall size of the research output and the volume of research activity is much smaller than that of public health and many other disciplines [20–22]. Previous literature has documented several challenges and barriers hindering the development of global health, such as shortage of funding opportunities, lack of societal and policy support, deficiency in global health workforce, and insufficient global experience [23, 24]. More efforts are needed to create a nurturing, collaborative environment for global health research to keep up the pace of China’s ambition to become a responsible and strong leader in global health.

We found that Chinese universities had a set of research priority areas. Of 19 research topics considered, NCDs were the most popular, accounting for over one-third of total publications. MCH and neurological and mental disorders and diseases were the second and third popular topics, accounting for 15.5% and 11.2% of all publications, respectively. A sizeable portion of publications also focused on infectious disease, health system and policy, and trauma and injury. However, the other two-thirds of the topics were under-researched, with fewer than 10 publications. We propose several plausible explanations for the predominant focus on NCDs research. First, China has experienced an epidemiological transition from infectious diseases to NCDs over a short period [25]. Deaths from infectious diseases have decreased by over 70% since 1990 in China, whereas the burden of NCDs has increased dramatically, accounting for nearly 90% of all deaths in recent years [26]. Prevention and control of NCDs and their modifiable risk factors have become one of the formidable challenges facing the healthcare system. The need to address NCDs through research is enormous. Second, NCDs and their risk factors are widely available in publicly accessible databases. Massive amount of NCDs data has been collected in a wide variety of research activities, including surveys, surveillance, and registries; many of these data sources are easily accessible electronically. For example, seven papers used data from the National Health and Nutrition Examination Survey to conduct epidemiological research on NCDs [27–33]. Third, the topic classification approach we adopted from the Fogarty International Center does not divide research areas into equally sized groups. Some topics, such as NCDs and MCH, are much more inclusive and broader than others (e.g., tobacco control). This might partially explain the huge difference in the number of publications between different research topics. While NCDs are one of many health challenges globally, more efforts are needed to diversify the global health research portfolios among global health institutions in China. As China has been increasingly becoming an aid donor and resource provider instead of an aid and funding recipient [34], strengthening research capacity in other global health areas is a critical step for China to broaden the scope and deepen the level of its global health engagement.

Global health research is versatile in methodology. The collection of primary data from the research field and secondary data analysis are the two most used approaches in original investigations. Of the 206 papers included, over 80% analyzed existing data, while < 20% involved primary data collection. Increasing access to and sharing health data is one driving force behind this phenomenon. On the other hand, researchers in China are hindered by the system, financial, and personal barriers to implementing field-based research with primary data collection oversea [1, 23]. Domestic funding agencies in China normally have strict policies and regulations on the administration of research grants. The allowable budget for conducting field-based research outside of China is limited, discouraging applications with a focus on global health. Compared to secondary data analyses, researchers are more likely to gain a deeper understanding of the research questions, design, procedures, and participants from field-based research. Field-based research provides an excellent opportunity to design and implement geopolitically and culturally relevant projects to tackle transnational health issues in real-world settings.

The limited availability of funding sources is another challenge to conducting global health research outside of China. Most of the funding for global health research conducted outside of China comes from private foundations and bilateral agencies, with China Medical Board and the UK Department for International Development being the two major contributors [23, 35]. Supporting health research outside of China has not been the priority for most, if not all, domestic funding agencies. International funding opportunities for Chinese institutions have gradually dwindled in recent years due to a variety of economic and geopolitical reasons. It is, therefore, necessary for China’s government funding agencies, such as the National Natural Science Foundation of China, to contribute more to global health research financially and logistically by launching new funding mechanisms. In addition, the current performance evaluation system in Chinese universities largely adopts a “publish or perish” principle. Faculty, especially those junior ones, lack the motivation to conduct field-based global health research, which requires a long-term commitment and a long investment cycle.

The importance of international collaborations to global health research cannot be overestimated. The Chinese government has made commitments to global health in recent years. There is still a lot to learn from countries that have pioneered the development of global health. For example, the UK has a long history of conducting global health research through its higher education institutions, non-government organizations, and international aid agencies [36]. The UK government has invested significantly in supporting global health research through national funding agencies, such as the Medical Research Council and the National Institute for Health Research. Similarly, the US has been a major player in global health through the establishment of international aid agencies and the provision of dedicated funding to support research. In this study, we found that scholars in Chinese global health institutions have collaborated with over 200 overseas institutions (i.e., primary affiliations of co-authors); a solid collaboration research network has been established. We noticed that these collaboration institutions were not evenly distributed globally, with over one-third and nearly 30% in North America and Asia, respectively. Country-specific results showed that the US had the highest number of collaboration institutions (n = 64; 28.7%). Collaborators from these US institutions mainly served as co-authors on papers analyzing secondary data. The size of research collaboration with institutions in low- and middle-income countries (LMICs) is relatively small, especially in Africa. These results suggest that profound political and economic relations with African countries do not naturally help identify research partners or establish research collaborations. Trilateral and multilateral platforms that expand from an existing collaboration between institutions in China and high-income countries to include other LMICs particularly African countries, are one feasible solution to overcome barriers to nurturing collaborations [10].

Global health research, education, and practice has been primarily based in schools of public health in China [37]. Relevant disciplines, such as public health and health management, have a much longer history in China. The Chinese government has made tremendous investments in public health infrastructure and there are a large number of Chinese universities offering degree and non-degree programs in public health and health management. Nevertheless, the pace of development for global health, a relatively young discipline, has accelerated recently in China with government support and a growing national emphasis on health and healthcare issues around the globe.

We acknowledge several limitations. First, authorship order and affiliation are imperfect measures of research capacity. However, the academic evaluation system in China has a long tradition of valuing primary affiliation and first/corresponding author of research publications. We, therefore, adopted a stringent criterion for selecting articles based on first/corresponding author to reflect the research contribution and capacity more accurately. Second, this study is by no means a thorough investigation of Chinese universities' research capacity in the global health field. We only considered articles published by ten founding members of the CCUGH. We might miss important trends in research conducted outside universities and emerging global health research institutions. Nevertheless, the ten founding members are the pioneers and leaders of global health research in China. They likely represent the general trends in global health research in China. Third, we did not include studies conducted entirely in China, which, strictly speaking, are also part of global health research. Our goal is to understand the trend and patterns of Chinese universities’ contributions to global health research (outside of China). Fourth, our study found that the number of peer-reviewed publications by the founding members of the CCUGH has steadily increased since 2014. These data suggest that engagement and research productivity in global health has improved. However, we could not rule out the possibility that the increase in research output results from the growing faculty size in these institutions. Caution is needed when interpreting these findings. Fifth, the present study adopted the Fogarty International Center’s classification to guide the categorization of global health research topics. We used this approach out of many choices primarily because it covers a wide range of global health topics and could easily classify majority of eligible papers identified in our study.

## Conclusions

In summary, despite a variety of challenges and barriers, Chinese universities have been playing an increasingly important role in global health research as assessed by peer-reviewed publications from 2014 to 2020 of original international research studies of the ten founding members of CCUGH. We also identified important gaps in current trends. More work is needed to diversify the global health research portfolios, promote investment in field-based research, and strengthen research collaborations with institutions in LMICs. Our study, although not exhaustive, provides a practical basis for planning and evaluating priority areas in global health research for Chinese universities. More concerted efforts by multiple stakeholders, including government, private sectors, funding agencies, academic institutions, and researchers, are needed to advance the development of global health research in China.

## Supplementary Information

**Additional file 1: Appendix 1:** Search strategy for each of the ten founding members in the Chinese Consortium of Universities for Global Health.

## Data Availability

Data are extracted from open access research databases. Summary data are available upon request.
