# Supplementary material for: Trends in global health research among universities in China: a bibliometric analysis
Source: Glob Health Res Policy. 2023 Apr 6;8:10. doi: 10.1186/s41256-023-00295-1 (PMC10078049; doi:10.1186/s41256-023-00295-1)
Supplement: Supplementary file 1 — Additional file 1: Appendix 1: Search strategy for each of the ten founding members in the Chinese Consortium of Universities for Global Health. [file 41256_2023_295_MOESM1_ESM.docx]

**Appendix 1:** Search strategy for each of the ten founding members in the Chinese Consortium of Universities for Global Health

**Central South University**

- **PubMed**: ("Central South University"[Affiliation] AND "XiangYa School of Public Health"[Affiliation]) AND 2014/01/01:2020/12/31[Date - Publication] AND ((journalarticle[Filter]) AND (humans[Filter]) AND (english[Filter]))
- **EMBASE:** ('Central South University':ff AND 'XiangYa School of Public Health':ff) AND 'human'/de AND 'article'/it AND [2014-2020]/py

**Duke Kunshan University**

- **PubMed:** ("Duke Kunshan University"[Affiliation] AND "Global Health Research Center"[Affiliation]) AND 2014/01/01:2020/12/31[Date - Publication] AND ((journalarticle[Filter]) AND (humans[Filter]) AND (english[Filter]))
- **EMBASE:** ('Duke Kunshan University':ff AND 'Global Health Research Center':ff) AND 'human'/de AND 'article'/it AND [2014-2020]/py

**Fudan University**

- **PubMed:** ("fudan university"[Affiliation] AND ("school of public health"[Affiliation] OR "global health institute"[Affiliation]) AND 2014/01/01:2020/12/31[Date - Publication]) AND ((journalarticle[Filter]) AND (humans[Filter]) AND (english[Filter]))
- **EMBASE:** 'fudan university':ff AND ('school of public health':ff OR 'global health institute':ff) AND 'human'/de AND 'article'/it AND [2014-2020]/py

**Kunming Medical College**

- **PubMed:** ("Kunming Medical University"[Affiliation] AND "school of public health"[Affiliation]) AND 2014/01/01:2020/12/31[Date - Publication] AND ((journalarticle[Filter]) AND (humans[Filter]) AND (english[Filter]))
- **EMBASE:** ('Kunming Medical University':ff AND 'school of public health':ff) AND 'human'/de AND 'article'/it AND [2014-2020]/py

**Peking University**

- **PubMed:** (("Peking University"[Affiliation] AND "school of public health"[Affiliation]) OR "Peking University Institute of Global Health and Development "[Affiliation]) AND 2014/01/01:2020/12/31[Date - Publication] AND ((journalarticle[Filter]) AND (humans[Filter]) AND (english[Filter]))
- **EMBASE:** (('peking university':ff AND 'school of public health':ff) OR 'peking university institute of global health and development':ff) AND 'human'/de AND 'article'/it AND [2014-2020]/py

**Peking Union Medical College**

- **PubMed:** ("Peking Union Medical College"[Affiliation] AND "school of public health"[Affiliation]) AND 2014/01/01:2020/12/31[Date - Publication] AND ((journalarticle[Filter]) AND (humans[Filter]) AND (english[Filter]))
- **EMBASE:** ('Peking Union Medical College':ff AND 'school of public health':ff) AND 'human'/de AND 'article'/it AND [2014-2020]/py

**Sun Yat-sen University**

- **PubMed**: ("Sun Yat-sen University"[Affiliation] AND "School of Public Health"[Affiliation]) AND 2014/01/01:2020/12/31[Date - Publication] AND ((journalarticle[Filter]) AND (humans[Filter]) AND (english[Filter]))
- **EMBASE:** ('Sun Yat-sen University':ff AND 'School of Public Health':ff) AND 'human'/de AND 'article'/it AND [2014-2020]/py

**The Chinese University of Hong Kong**

- **PubMed:** ("The Chinese University of Hong Kong"[Affiliation] AND ("The Jockey Club School of Public Health and Primary Care"[Affiliation] OR "Centre for Global Health"[Affiliation]) AND 2014/01/01:2020/12/31[Date - Publication]) AND ((journalarticle[Filter]) AND (humans[Filter]) AND (english[Filter]))
- **EMBASE:** 'The Chinese University of Hong Kong':ff AND ('The Jockey Club School of Public Health and Primary Care':ff OR 'Centre for Global Health':ff) AND 'human'/de AND 'article'/it AND [2014-2020]/py

**Wuhan University**

- **PubMed:** ("Wuhan University"[Affiliation] AND ("school of public health"[Affiliation] OR "School of Health Sciences"[Affiliation] OR "global health institute"[Affiliation])) AND 2014/01/01:2020/12/31[Date - Publication] AND ((journalarticle[Filter]) AND (humans[Filter]) AND (english[Filter]))
- **EMBASE:** ('Wuhan University':ff AND ('school of public health':ff OR School of Health Sciences':ff OR 'global health institute':ff)) AND 'human'/de AND 'article'/it AND [2014-2020]/py

**Zhejiang University**

- **PubMed**: ("Zhejiang University"[Affiliation] AND "school of public health"[Affiliation]) AND 2014/01/01:2020/12/31[Date - Publication] AND ((journalarticle[Filter]) AND (humans[Filter]) AND (english[Filter]))
- **EMBASE:** ('Zhejiang University':ff AND 'school of public health':ff) AND 'human'/de AND 'article'/it AND [2014-2020]/py
